# Genetic crosstalk of autism spectrum disorders and epilepsy: an insight into the presynapse

**DOI:** 10.3389/fneur.2025.1677134

**Published:** 2025-11-03

**Authors:** Mahima Sharma, Sai Charan Pamidi, Pavan Kumar Divi, Saswati Mohapatra, Brijit George, Karen P. Sneha, Judith C. Kreutzmann, Anil Annamneedi

**Affiliations:** ^1^Department of Biotechnology, Faculty of Engineering and Technology, SRM Institute of Science and Technology, Chennai, Tamil Nadu, India; ^2^Department of Medical Biochemistry and Biophysics, Karolinska Institutet, Stockholm, Sweden; ^3^School of Arts and Sciences, Sai University, Chennai, Tamil Nadu, India

**Keywords:** ASD, epilepsy, presynaptic genes, comorbidity, abnormal synaptic plasticity, synaptic organization

## Abstract

The neurodevelopmental disorder autism spectrum disorder (ASD) affects 0.5%–1% of the global population and is marked by ongoing difficulties in social communication and cognitive function. Interestingly, ASD has been reported to share a genetic origin with epilepsy, a condition marked by recurrent, unprovoked seizures. Both ASD and epilepsy are caused by multifactorial and multigenetic origin. Whereas the number of genes linked to ASD etiology are growing, the genetic basis of epilepsy is more diverging leading to distinct epileptic syndromes. Despite decades of discussion, a comprehensive understanding of the genetic interplay between these disorders remains elusive. Our article focuses on investigating the shared genetic basis of abnormalities in synaptic proteins, highlighting the presynaptic compartment, which is less explored compared to the postsynaptic elements. We identify those biological processes linked to the presynaptic compartment, such as presynaptic assembly, ATP metabolism, various aspects of the synaptic vesicle cycle, are commonly affected across conditions, as evidenced by the shared genetics. Hence, this study offers initial insights into presynaptic signaling, and further research could aid in developing improved therapeutic strategies by targeting these presynaptic processes.

## 1 Introduction

Autism spectrum disorder (ASD) is a neurodevelopmental disorder that typically manifest at birth, persisting throughout an individual’s life causing behavioral, cognitive, and social challenges. Epilepsy, on the other hand, can occur throughout the life span of an individual due to various other neurological compromises including stroke, tumors or other pathologies. Notably, epilepsy is a common comorbid condition in individuals with ASD. Although both environmental and genetic factors contribute to the co-occurrence of ASD and epilepsy (1), genetic factors play a predominant role in the development of these disorders. Recent studies highlight that the co-occurrence of ASD and epilepsy is largely driven by disruptions in fundamental neurodevelopmental pathways. Shared genetic mutations affecting ion channels, synaptic proteins, and transcription factors contribute to these disruptions, leading to altered neural connectivity and excitability that underlie both autistic behaviors and epileptic seizures (2). Approximately 10%–20% of individuals with ASD share genetic factors that overlap with epilepsy (3). Interestingly, about 30% of individuals diagnosed with epilepsy also meet certain diagnostic criteria for ASD (4, 5). Notably, epilepsy may potentially contribute to the development of ASD, or conversely, the abnormal brain circuitry underlying ASD could predispose individuals to epileptic seizures.

The genetic causes of ASD and epilepsy involve dysregulation of synaptic functions due to mutations in genes such as *SYN1* (synapsin-1), *SCN2A*, and *SCN8A* (sodium voltage-gated channel alpha subunit 2 and 8), *KCNQ2* and *KCNQ5* (potassium voltage-gated channel subfamily Q member 2 and 5), *SHANK3* (glutamate receptor signaling protein SH3 and multiple ankyrin repeat domains 3), *GABRG2* or *GABRG3* (gamma-aminobutyric acid type A receptor gamma subunits 2 and 3). These genes are typically linked to synaptic compartments, and span across the pre- and post-synapse (6–9). However, despite its critical roles in neurotransmitter maintenance and release, neural circuit development, and activity regulation, our understanding of the presynaptic compartment in relation to neuropathology remains elusive. Although many genes are shared between ASD and epilepsy, the effects of specific regulatory mutations—such as loss- or gain-of-function variants—on disease onset and severity remain poorly understood. This highlights the need for closer examination of the functional consequences of these variants.

Current knowledge of proteins localized to the presynaptic active zone, such as *RIM* (Rab3A-interacting molecule), *RIM-BP* (RIM-binding protein), *BSN* (Bassoon), *PCLO* (Piccolo), *PPFIA1* (Liprin-*α*), is limited despite their crucial roles. These active zone-specific scaffolding molecules have been associated with various conditions including ASD, intellectual disability, epilepsy, or schizophrenia (10–16). Their implications in these diseases underscore their significant impact on synaptic transmission and circuit development.

In this study, we investigate the genetic associations between ASD and epilepsy, specifically exploring the signaling pathways mediated by presynaptic genes. Our objective is to shed light on potential alterations in presynaptic and overall synaptic functions, thereby characterizing the presynaptic compartment as a target for novel therapeutic drug interventions. By conducting a systematic literature review and employing subsequent synaptic enrichment analysis using the SynGO database, we identified common genes associated with both ASD and epilepsy, highlighting a significant subset of synaptic genes. Beyond cataloging shared genes, our analysis specifically focuses on the nature of identified variants (loss- versus gain-of-function) and their mechanistic impact on synaptic processes. This approach provides a more comprehensive understanding of the genetic and functional interplay underlying ASD and epilepsy comorbidity. Moreover, characterizing variants as loss- or gain-of-function will help identifying promising candidates for precision therapies targeting synaptic dysfunctions.

## 2 Methods

A list of genes linked to both ASD and epilepsy was compiled through an extensive literature search on google scholar and PubMed using the keywords “comorbidity of ASD and epilepsy”; “ASD in epilepsy”; “Epilepsy percentage in ASD”; “genetics of ASD and epilepsy” and from the databases for ASD (SFARI: https://gene.sfari.org/database/human-gene/), epilepsy (EpilepsyGene: http://www.wzgenomics.cn/EpilepsyGene/index.php; epiGAD: https://www.epigad.org/index.html; CarpeDG: http://carpedb.ua.edu/search.cfm). Common genes implicated in both diseases were compiled, and a list of associated synaptic genes was identified using the synaptic gene ontologies (SynGO) database (17). Using the domain ‘Cellular Components’ (location), genes localized to the presynaptic region were identified. Their involvement in various processes was further identified and focused on by using the domain ‘Biological Process’ (Supplementary Figure 1).

## 3 Results and discussion

In our SynGO analysis, we identified 49 synaptic genes out of 125 common genes (Supplementary Table 1). Among these, 16 genes are exclusively associated with presynaptic localization and function, while 19 genes are linked to postsynaptic roles. Employing SynGO enrichment analysis, we further identified several synaptic genes based on the localization and biological processes that are common to both ASD and epileptic phenotypes (Supplementary Figure 2A and Supplementary Table 2). Additionally, 14 genes are shared between the pre- and post-synapse (Supplementary Figure 2A). Based on this analysis, the presynaptic genes are specifically localized to various cellular components (Supplementary Figure 2B).

**Figure 1:**
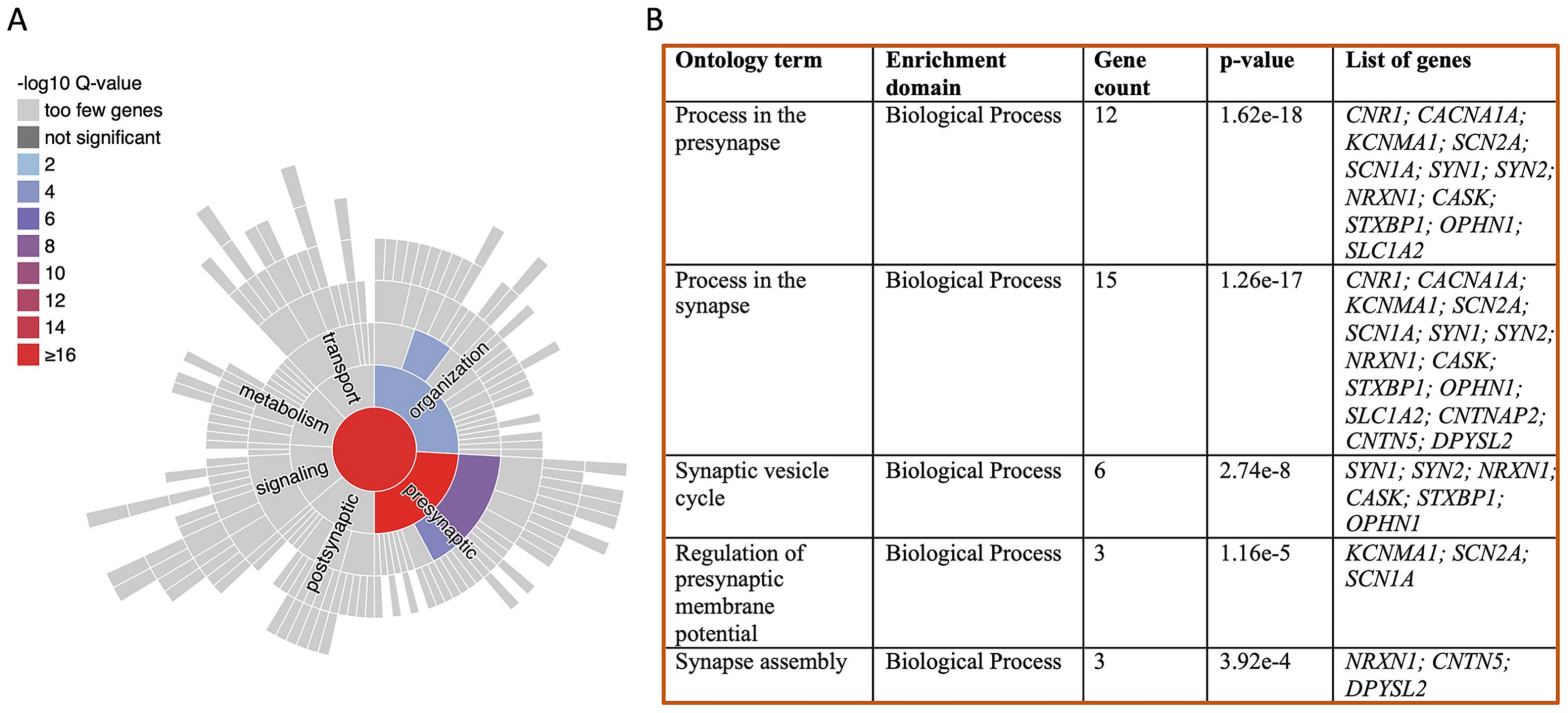
**(A)** Sunburst image depicts the gene enrichment analyses for common synaptic genes associated with ASD and epilepsy, categorized by biological processes. **(B)** Summary of the SynGO gene ontology database, categorizing gene products based on their biological processes and the functional processes that they are linked to. Key process within the presynapse, such as the synaptic vesicle cycle and regulation of membrane potential, show significant enrichment. Additionally, processes related to synapse organization indicating the disruptions in overall synaptic function in both ASD and epilepsy, primarily originating from the presynaptic compartment.

The list of identified common ASD-epilepsy genes localized at the presynaptic compartment are involved in various synaptic processes, including synaptic assembly, regulation of presynaptic processes, synaptic signaling, and metabolism (Figures 1A,B). Mutations in these associated genes or resulting protein dysfunctions have been shown to impact these processes during the progression of ASD and epilepsy.

**Figure 2:**
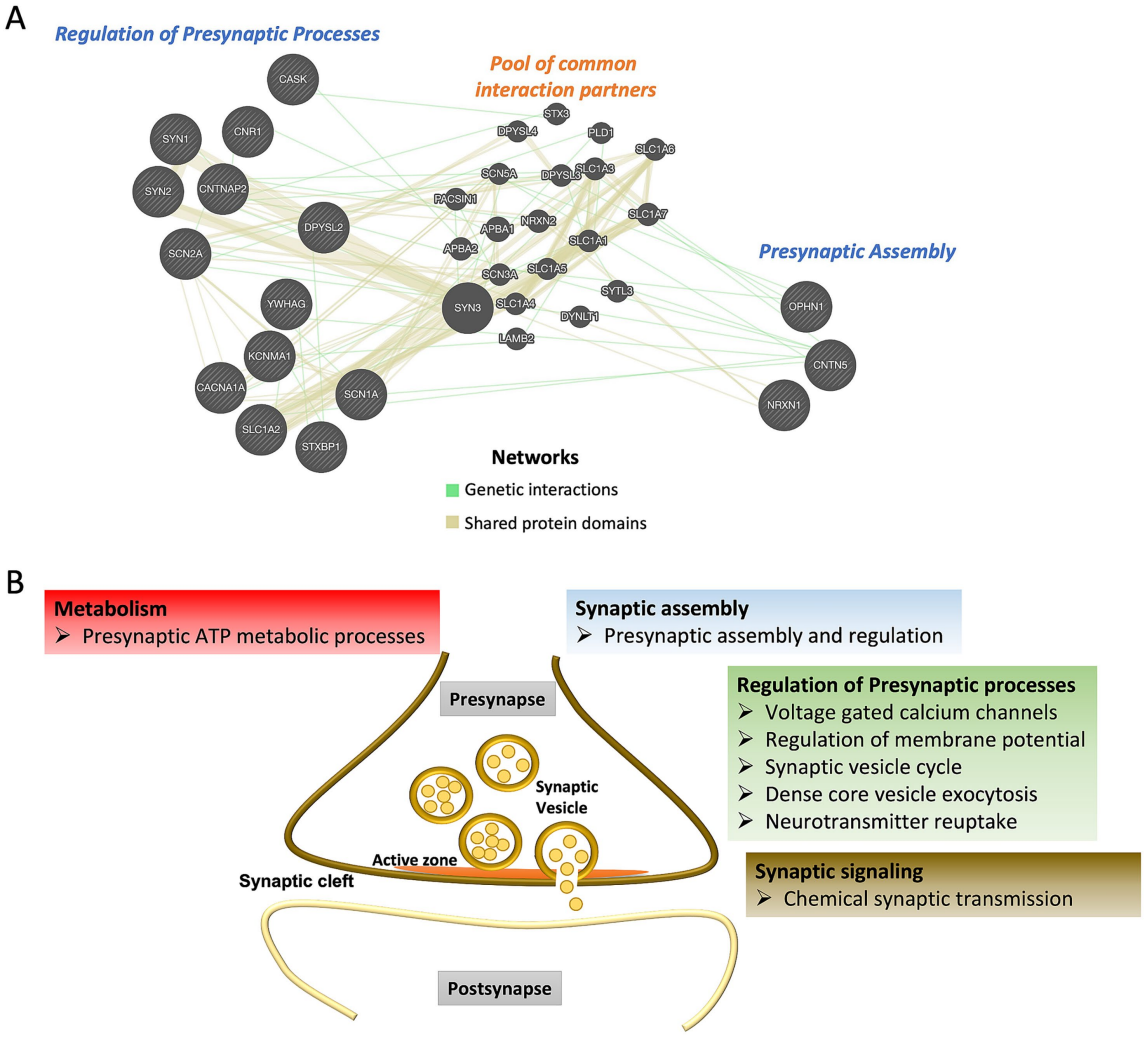
**(A)** Interactions among the shared ASD and epilepsy presynaptic genes regulating different biological functions. GeneMANIA database was used to identify interaction partners. **(B)** Schematic representation of presynaptic processes linked to common genes for ASD and epilepsy.

There are nearly 40% of genes associated with both ASD and epilepsy are synaptic genes, as identified through a gene ontology study using SynGO and the presynaptic function is as crucial as postsynaptic function in disease pathogenesis. While much attention has been devoted to understanding the postsynaptic receptor signaling in disease progression and drug development, knowledge about the presynaptic compartment remains limited. Our analysis underscores the significant enrichment of various processes within the presynaptic compartment. Disruption of these processes could have profound impact on overall synaptic function (Figure 2B), highlighting the critical need to investigate presynaptic mechanisms for a comprehensive understanding of disorders.

The *Presynaptic Assembly* involves three major steps: neuronal contact formation, synaptic precursor transport, and the cessation of transport processes at the contact sites. The *CNTN5* gene encodes the protein Contactin-5, a member of the immunoglobulin superfamily of cell adhesion molecules critical for nervous system development, particularly in axonal contact formation. *CNTN5* is primarily expressed postnatally in the central nervous system, including the cerebral cortex (auditory cortex), thalamus, and caudate putamen (18). Gene mutations or copy number variation (CNVs) in *CNTN5* have been linked to ASD and epilepsy (19, 20). Loss of *CNTN5* leads to synaptic dysfunction, resulting in heightened neuronal excitability (21).

*OPHN1* encodes Oligophrenin-1, a Rho-GTPase-activating protein (RhoGAP) expressed ubiquitously in the developing brain. Oligophrenin-1 functions as extracellular growth and guiding signal mediators important for the linking of these signals originated from the cell-surface adhesion molecules to the intracellular signal transduction pathways. These pathways are crucial for neuronal morphogenesis, and cytoskeletal dynamics by orienting the actin molecules at axonal growth cones (22, 23). Deletion or mutations in *OPHN1* are associated with nonspecific X-linked intellectual disability, ASD, intellectual disability, epilepsy, enlargement of ventricles in the brain, ataxia, and cerebellar hypoplasia (24). Loss of *OPHN1* function results in impaired maturation of dendritic spines (25).

The *NRXN1* gene encodes Neurexin 1, a presynaptically localized membrane protein involved in the formation of Ca^2+^-dependent surface receptor complexes. Neurexins form complexes with neuroligins, facilitating efficient synaptic contact formation and neurotransmission by linking calcium (Ca^2+^) channels to synaptic vesicles for exocytosis (26). The expression of different neurexins occurs during early cortical plate formation before extensive synaptogenesis takes place, with age-dependent increase in the expression of Neurexin1 (27). Mutations in the human *NRXN1* gene have been implicated in several neuropathological conditions, including ASD, schizophrenia, autosomal recessive intellectual disability, Pitt-Hopkins-like syndrome, attention-deficit hyperactivity disorder (ADHD), and epilepsy (28, 29). Loss-of-function mutations in the *NRXN1* gene disrupt protein–protein interactions, leading to synaptic dysfunctions, whereas gain-of-function mutations promote increased excitatory synaptogenesis and neuronal excitability, potentially via enhanced calcium signaling (30–32).

The *Regulation of Presynaptic Processes,* such as maintaining Ca^2+^ levels, ion channel activity to balance the membrane potential, and the synaptic vesicle cycle (encompassing exocytosis and neurotransmitter reuptake), is mediated by several proteins at the presynaptic terminal. The list of identified presynaptic genes from our analysis falls under the processes mentioned above that occur at the presynaptic compartment. *CACNA1A* encodes the α1A pore-forming subunit of the voltage-gated P/Q-type calcium channel (Cav2.1), which mediates its function at the presynaptic terminal (33). These channels are widely expressed throughout the central nervous system and are particularly abundant in brain regions such as the cerebellum, especially in Purkinje and granule cells (34). The Cav2.1 channel facilitates synaptic vesicle exocytosis through Ca^2+^-influx, thus playing a crucial role in neurotransmission. Haploinsufficiency or *de novo* mutations in the *CACNA1A* gene can lead to the development of epileptic encephalopathy, ASD, and schizophrenia (35).

The *CNR1* gene encodes the type 1 cannabinoid receptor (CB1), which is part of the endocannabinoid system and is the receptor for the most widely used yet controversial psychoactive drug, cannabis. *CNR1* expression is higher during the fetal stage compared to the postnatal stage in various brain areas, such as the prefrontal cortex, hippocampus, and caudate. The CB1 receptor, a G-protein-coupled receptor, is expressed presynaptically on neuronal terminals in brain regions including the hippocampus, amygdala, hypothalamus, midbrain, frontal cortex, and cerebellum, where it regulates the gamma-aminobutyric acid (GABA)ergic and glutamatergic transmission (36, 37). Genetic variations in the *CNR1* gene are associated with neurological disorders, including ASD (38).

The Potassium Calcium-Activated Channel Subfamily M Alpha 1, encoded by *KCNMA1* gene and commonly referred to as the Big K + (BK) channel exhibits exceptionally high conductance (>100 pS). These channels are predominantly expressed in the brain and muscle tissues and are classified within the voltage-gated K + channel family. BK channels are recognized for their ability to respond to changes in voltage, thereby regulating excitability through mediating potassium efflux, alongside intracellular calcium levels, making them pivotal in regulating neuronal and muscular function. Dysfunction or loss of BK channel can result from mutations or single nucleotide polymorphisms (SNPs) in the *KCNMA1* gene. Such genetic alterations have been implicated in various disorders including autism, intellectual disability, epilepsy, hypertension, asthma (39, 40).

*SCN1A* and *SCN2A* encode the alpha subunit of the voltage-gated sodium channels Nav1.1 Nav1.2 (41). Both channels are expressed in the central nervous system and function as transmembrane protein complexes composed of glycosylated alpha subunits that form ion-conducting pores. Together, they play a crucial role in sodium exchange, as well as action potential generation and propagation among neurons, thus regulating excitability. Nav1.1 and Nav1.2 are widely distributed across the cerebral cortex, hippocampal CA3 and CA2 regions, dentate gyrus, thalamus, substantia nigra, putamen and cerebellum (42). *SCN1A* and *SCN2A* are considered risk genes for ASD due to their proximity to autism susceptibility loci on chromosomes (43). Additionally, mutations in these genes are associated with various forms of seizures, such as generalized epilepsy with febrile seizures plus or myoclonic epilepsy (44). Loss of *SCN1A* impairs inhibitory neuron excitability, leading to Dravet syndrome and ASD-like features, whereas gain-of-function mutations contribute to early-onset epilepsy and familial hemiplegic migraine type 3 (FHM3) (45). Similarly, gain-of-function variants in *SCN2A* are associated with early-infantile epilepsies (seizure onset before 3 months of age), while loss-of-function variants result in late-onset epilepsies and ASD/ID (46).

Synapsin family proteins, such as Synapsin1 encoded by *SYN1* and Synapsin2 encoded by *SYN2*, are phosphoproteins that bind to synaptic vesicles (SVs). They are essential for neurotransmitter release and synaptic plasticity by participating in various steps of the SV cycle, including SV tethering, docking, fusion. These proteins also play an important role in synaptogenesis and have been implicated to be involved in key aspects of neuronal development, axonogenesis, and synaptic maintenance (47). As *SYN1* and *SYN2* are X-linked genes, mutations in these genes are associated to X-linked neurodevelopmental disorders, primarily affecting males with clinical presentation of epilepsy, learning disabilities, etc. Additionally, genetic variants in *SYN1* and *SYN2* are linked to ASD traits and X-linked intellectual disability across various ethnic backgrounds (47, 48). Mutations in *SYN1* impair neurotransmitter release, neurite outgrowth, and synaptic vesicle pool trafficking (47, 49). Similarly, the loss-of-function mutations in *SYN2* produce phenotypes nearly identical to those observed with *SYN1* variants (48).

Calcium/Calmodulin Dependent Serine Protein Kinase (*CASK*) is a protein-coding gene belonging to the MAGUK (membrane-associated guanylate kinase) family of proteins and is ubiquitously expressed in the developing brain. At the presynaptic compartment, *CASK* regulates SV exocytosis, interacts with *NRXN1*, and contributes to maintaining the excitatory/inhibitory (E/I) balance by modulating ionotropic receptor trafficking (50). Located on the X-chromosome, loss of CASK is associated with X-linked intellectual disability, ASD and epilepsy (13). Recent studies have shown that loss-of-function mutations in *CASK* result in distinct phenotypes, including impaired neuronal outgrowth during development and reduced excitability during adulthood (51).

The *STXBP1* gene encodes syntaxin-binding protein 1 (also known as MUNC18-1), which plays a role in neurotransmitter release by participating in SV cycle steps such as docking, priming and fusion through interactions with SNARE proteins (52). *De novo* heterozygous mutations in *STXBP1* lead to severe forms of epileptic encephalopathies, including Ohtahara syndrome or Dravet syndrome (53). Mutations in the *STXBP1* gene have been linked to intellectual disability and other neurodevelopmental conditions, such as ASD (54). While loss of *STXBP1* leads to presynaptic dysfunction, neurodegeneration, and hyperexcitability (55, 56), gain-of-function mutations enhance synaptic functions (57).

The *SLC1A2* gene encodes Solute Carrier Family1 Member2 (EAAT2), a member of the solute transporter protein family. *SLC1A2* is responsible for clearing glutamate from the extracellular space between synapses and facilitates its reuptake to maintain excitatory neurotransmission. EAAT2 is the predominant glutamate transporters in the brain, accounting for over 95% of total glutamate uptake activity (58). Mutations in the *SLC1A2* gene are primarily associated with epileptic encephalopathy, with some reports also linking them to ASD and intellectual disability (59). Mutations in *SLC1A2* cause glutamate dysregulation, disrupted Ca^2+^ storage in the endoplasmic reticulum, and reduced EAAT2 expression and glutamate transport (60). Mild gain-of-function variants of *SLC1A2* lead to modest increases in anion currents (61).

The *CNTNAP2* gene, primarily active during the brain development, encodes the single-pass transmembrane protein contactin-associated protein-like 2 (CASPR2) protein. As a member of cell adhesion molecules, such as the neurexin superfamily, CASPR2 is crucial for synapse formation, neurite outgrowth and myelination through its interaction with contactin-1. The expression of *CNTNAP2* is restricted to specific regions of the brain, including the cortex, striatum, and thalamus, thereby participating in the regulation of higher cognitive functions. Loss-of-function mutations in *CNTNAP2* disrupt excitatory neuron development, reduce neurite branching and neuronal complexity, and impair cortical connectivity, contributing to intellectual disability, ASD, epilepsy, schizophrenia, and depression (62–65).

Dihydropyrimidinase-protein 2, also known as Collapsin response mediator protein-2, is encoded by the *DPYSL2* gene and is crucial for neuronal development, cell migration and axonal growth and guidance, thus contributing to neuronal polarity. Dihydropyrimidinase-protein 2 is also involved in synaptic transmission, calcium homeostasis, neurotransmitter release, cytoskeletal dynamics and vesicle trafficking (66). Polymorphisms or mutations in *DPYSL2* are associated with schizophrenia, intellectual disability, and epilepsy (67, 68). Loss of *DPYSL2* leads to defects in axonal pruning and corpus callosal axon guidance (69).

The *YWHAG* gene encodes the adapter protein 14–3-3 protein gamma, a member of the 14–3-3 protein family, which is ubiquitously expressed in brain. 14–3-3 proteins bind to various other proteins containing phosphoserine sites and are involved in neuronal migration by mediating signal transduction. Through interactions with presynaptic active zone proteins, 14–3-3 regulates presynaptic remodeling during synaptic plasticity and long-term potentiation (70). *De novo* missense mutations in *YWHAG* are linked to epileptic encephalopathies, ASD and intellectual disability (71, 72).

Interactions among these genes (also known as epistasis) or the end products-proteins is responsible for physiological functions as well drive the complexity of disease pathology. List of identified shared presynaptic genes display interactions among and in between the biological processes arguing for a crosstalk among different functional aspects and synergistic approach in mediating the crucial synaptic functions (Figure 2A and Supplementary Table 3).

In conclusion, our analysis highlights the critical role of presynaptic signaling, which can be disrupted by mutations in genes commonly associated to both ASD and epilepsy. While the relationship between these two disorders has been described for decades, substantial evidence for a shared mechanistic basis underlying their core symptoms and for the efficacy of therapeutic intervention remains limited. Emerging data suggest that dysfunction of presynaptic genes is a key contributor to disease progression in both conditions. So far, a handful of studies have highlighted that targeting specific presynaptic components, such as receptors regulating neurotransmitter release or kinases essential for axonal transport, may offer promising avenues for pharmacological interventions (73, 74). Nevertheless, a more in-depth investigation into presynaptic signaling pathways and mechanisms mediating various presynaptic processes and assembly (Figure 2B) could provide additional targets for novel therapeutics. Future interventions should carefully consider the functional consequences of diverse gene mutations, including gain- and loss-of-function variants, to enable precision therapeutics for these comorbidities.

## Data Availability

The original contributions presented in the study are included in the article/Supplementary material, further inquiries can be directed to the corresponding author.
