# Supplementary material for: Genetic crosstalk of autism spectrum disorders and epilepsy: an insight into the presynapse
Source: Front Neurol. 2025 Nov 3;16:1677134. doi: 10.3389/fneur.2025.1677134 (PMC12620427; doi:10.3389/fneur.2025.1677134)
Supplement: Supplementary file 1 [file Data_Sheet_1.pdf]

Genetic crosstalk of autism spectrum disorders and epilepsy: an insight into the presynapse

Supplementary Material

1 Supplementary Tables

Supplementary table 1: The total list of common genes identified between ASD and epilepsy.

List of common genes (ASD-Epilepsy)

ABAT	GABBR2	RALGAPB
ABL2	GABRB2	RANBP17
ADNP	GABRB3	RBFOX1
ADSL	GLRA2	RELN
AGTR2	GRIN1	RFX3
ALDH5A1	GAS2	SCAF4
ALG6	GRIN2A	SCN1A
AMT	GRIN2B	SCN2A
ANK3	HCN1	SCN8A
ARHGEF9	HDAC4	SCN9A
ARX	HNRNPU	SLC1A1
ASH1L	IQSEC2	SLC1A2
ATP10A	ITPR1	SLC25A12
BCKDK	KCNB1	SLC6A4
BRAF	KCND2	SLC6A8
CACNA1A	KCNJ10	SLC9A6
CACNA1E	KCNMA1	SMURF1
CACNA1G	KCNQ2	STXBP1
CACNA1H	KCNQ3	SYN1
CACNA1I	KMT2A	SYN2
CAMK4	MAOA	SYNGAP1
CASK	MAPK8IP1	TAF1
CDKL5	MAPT	TCF4
CHD2	MECP2	TRIM32
CHRNA7	MEF2C	TRIO
CLCN4	MTHFR	TRRAP
CLN8	MTOR	TSC1
CNR1	MYO5A	TSC2
CNTN5	MYO9B	TTN
CNTNAP2	NBEA	UBE3A
CREBBP	NF1	UBR5
CSMD3	NIPA1	WWOX
CSNK1E	NIPA2	XPO1
CSNK1G1	NLGN2	YWHAG
CUX1	NR2F1	ZMYND8

CUX2	NRXN1
DEPDC5	OPHN1
DIP2C	PACS2
DPYSL2	PAX6
DYRK1A	PCDH19
EEF1A2	PRICKLE1
ELP4	PRICKLE2
EN2	PRODH
EPHB1	PTEN
FMR1	PTGS2

Supplementary table 2: Table displaying the SynGO analysis of synaptic genes and the respective biological processes domain.

SynGO analysis_GO domain: Biological Processes (BP)					
gene hgnc_id	gene symbol	gene name	GO term ID	GO term name	SynGO annotation ID
HGNC:11444	STXBP1	syntaxin binding protein 1	GO:0016082	synaptic vesicle priming (GO:0016082)	82
HGNC:14561	ARHGEF9	Cdc42 guanine nucleotide exchange factor 9	GO:0099150	regulation of postsynaptic specialization assembly (GO:0099150)	156
HGNC:6180	ITPR1	inositol 1,4,5-trisphosphate receptor type 1	GO:0098695	inositol 1,4,5-trisphosphate receptor activity involved in regulation of postsynaptic cytosolic calcium levels (GO:0098695)	159
HGNC:3775	FMR1	FMRP translational regulator 1	GO:0099577	regulation of translation at presynapse, modulating synaptic transmission (GO:0099577)	199
HGNC:3775	FMR1	FMRP translational regulator 1	GO:0099578	regulation of translation at postsynapse, modulating synaptic transmission (GO:0099578)	200
HGNC:1497	CASK	calcium/calmodulin dependent serine protein kinase	GO:2000300	regulation of synaptic vesicle exocytosis (GO:2000300)	233
HGNC:11494	SYN1	synapsin I	GO:0098693	regulation of synaptic vesicle cycle (GO:0098693)	318
HGNC:1388	CACNA1A	calcium voltage-gated channel subunit alpha1 A	GO:0099626	voltage-gated calcium channel activity involved in regulation of presynaptic cytosolic calcium levels (GO:0099626)	411

HGNC:6990	MECP2	methyl-CpG binding protein 2	GO:0050807	regulation of synapse organization (GO:0050807)	492
HGNC:10588	SCN2A	sodium voltage-gated channel alpha subunit 2	GO:0099508	voltage-gated ion channel activity involved in regulation of presynaptic membrane potential (GO:0099508)	533
HGNC:11055	SLC6A8	solute carrier family 6 member 8	SYNGO:metabolism	metabolism	775
HGNC:2159	CNR1	cannabinoid receptor 1	SYNGO:presyn_regulationATP metabolic	regulation of ATP metabolic process in the presynapse	795
HGNC:2159	CNR1	cannabinoid receptor 1	GO:0099509	regulation of presynaptic cytosolic calcium levels (GO:0099509)	808
HGNC:11050	SLC6A4	solute carrier family 6 member 4	GO:0098810	neurotransmitter reuptake (GO:0098810)	819
HGNC:8008	NRXN1	neurexin 1	GO:0150036	regulation of trans-synaptic signaling by endocannabinoid, modulating synaptic transmission (GO:0150036)	895
HGNC:8008	NRXN1	neurexin 1	GO:0098693	regulation of synaptic vesicle cycle (GO:0098693)	898
HGNC:1464	CAMK4	calcium/calmodulin dependent protein kinase IV	GO:0099527	postsynapse to nucleus signaling pathway (GO:0099527)	925
HGNC:2175	CNTN5	contactin 5	GO:0099054	presynapse assembly (GO:0099054)	973

HGNC:29059	IQSEC2	IQ motif and Sec7 domain ArfGEF 2	GO:0098696	regulation of neurotransmitter receptor localization to postsynaptic specialization membrane (GO:0098696)	1057
HGNC:2159	CNR1	cannabinoid receptor 1	GO:0098921	retrograde trans-synaptic signaling by endocannabinoid (GO:0098921)	1066
HGNC:29059	IQSEC2	IQ motif and Sec7 domain ArfGEF 2	GO:0050804	modulation of chemical synaptic transmission (GO:0050804)	1077
HGNC:11495	SYN2	synapsin II	GO:0097091	synaptic vesicle clustering (GO:0097091)	1158
HGNC:11411	CDKL5	cyclin dependent kinase like 5	GO:0099175	regulation of postsynapse organization (GO:0099175)	1278
HGNC:4082	GABRB2	gamma-aminobutyric acid type A receptor subunit beta2	GO:0099507	ligand-gated ion channel activity involved in regulation of presynaptic membrane potential (GO:0099507)	1336
HGNC:10585	SCN1A	sodium voltage-gated channel alpha subunit 1	GO:0099508	voltage-gated ion channel activity involved in regulation of presynaptic membrane potential (GO:0099508)	1347
HGNC:4586	GRIN2B	glutamate ionotropic receptor NMDA type subunit 2B	GO:0099507	ligand-gated ion channel activity involved in regulation of presynaptic membrane potential (GO:0099507)	1351
HGNC:4585	GRIN2A	glutamate ionotropic receptor NMDA type subunit 2A	GO:0099507	ligand-gated ion channel activity involved in regulation of presynaptic membrane potential (GO:0099507)	1352

HGNC:4584	GRIN1	glutamate ionotropic receptor NMDA type subunit 1	GO:0099507	ligand-gated ion channel activity involved in regulation of presynaptic membrane potential (GO:0099507)	1353
HGNC:11497	SYNGAP1	synaptic Ras GTPase activating protein 1	GO:0098880	maintenance of postsynaptic specialization structure (GO:0098880)	1372
HGNC:6284	KCNMA1	potassium calcium-activated channel subfamily M alpha 1	GO:0099507	ligand-gated ion channel activity involved in regulation of presynaptic membrane potential (GO:0099507)	1446
HGNC:11494	SYN1	synapsin I	GO:0099504	synaptic vesicle cycle (GO:0099504)	1466
HGNC:11495	SYN2	synapsin II	GO:0099504	synaptic vesicle cycle (GO:0099504)	1467
HGNC:3014	DPYSL2	dihydropyrimidinase like 2	SYNGO:transport	transport	1628
HGNC:3392	EPHB1	EPH receptor B1	GO:0050804	modulation of chemical synaptic transmission (GO:0050804)	1738
HGNC:10940	SLC1A2	solute carrier family 1 member 2	GO:0098810	neurotransmitter reuptake (GO:0098810)	1820
HGNC:5048	HNRNPU	heterogeneous nuclear ribonucleoprotein U	GO:0098963	dendritic transport of messenger ribonucleoprotein complex (GO:0098963)	1834
HGNC:3942	MTOR	mechanistic target of rapamycin kinase	GO:0099547	regulation of translation at synapse, modulating synaptic transmission (GO:0099547)	1869
HGNC:8008	NRXN1	neurexin 1	GO:0099054	presynapse assembly (GO:0099054)	1934

HGNC:8008	NRXN1	neurexin 1	GO:0099151	regulation of postsynaptic density assembly (GO:0099151)	1935
HGNC:4584	GRIN1	glutamate ionotropic receptor NMDA type subunit 1	GO:1904315	transmitter-gated ion channel activity involved in regulation of postsynaptic membrane potential (GO:1904315)	1969
HGNC:4585	GRIN2A	glutamate ionotropic receptor NMDA type subunit 2A	GO:1904315	transmitter-gated ion channel activity involved in regulation of postsynaptic membrane potential (GO:1904315)	1970
HGNC:4586	GRIN2B	glutamate ionotropic receptor NMDA type subunit 2B	GO:1904315	transmitter-gated ion channel activity involved in regulation of postsynaptic membrane potential (GO:1904315)	1971
HGNC:1960	CHRNA7	cholinergic receptor nicotinic alpha 7 subunit	GO:1904315	transmitter-gated ion channel activity involved in regulation of postsynaptic membrane potential (GO:1904315)	1994
HGNC:1960	CHRNA7	cholinergic receptor nicotinic alpha 7 subunit	GO:0099171	presynaptic modulation of chemical synaptic transmission (GO:0099171)	2024
HGNC:4327	GLRA2	glycine receptor alpha 2	GO:1904315	transmitter-gated ion channel activity involved in regulation of postsynaptic membrane potential (GO:1904315)	2028
HGNC:4083	GABRB3	gamma-aminobutyric acid type A receptor subunit beta3	GO:1904315	transmitter-gated ion channel activity involved in regulation of postsynaptic membrane potential (GO:1904315)	2060
HGNC:7602	MYO5A	myosin VA	GO:0099089	establishment of endoplasmic reticulum localization to postsynapse (GO:0099089)	2143

HGNC:6238	KCND2	potassium voltage-gated channel subfamily D member 2	GO:1905030	voltage-gated ion channel activity involved in regulation of postsynaptic membrane potential (GO:1905030)	2159
HGNC:7602	MYO5A	myosin VA	SYNGO:transport	transport	2197
HGNC:7602	MYO5A	myosin VA	GO:0099566	regulation of postsynaptic cytosolic calcium levels (GO:0099566)	2244
HGNC:4082	GABRB2	gamma-aminobutyric acid type A receptor subunit beta2	GO:1904315	transmitter-gated ion channel activity involved in regulation of postsynaptic membrane potential (GO:1904315)	2253
HGNC:12363	TSC2	TSC complex subunit 2	GO:0099175	regulation of postsynapse organization (GO:0099175)	2415
HGNC:1392	CACNA1E	calcium voltage-gated channel subunit alpha1 E	GO:0099626	voltage-gated calcium channel activity involved in regulation of presynaptic cytosolic calcium levels (GO:0099626)	2419
HGNC:1392	CACNA1E	calcium voltage-gated channel subunit alpha1 E	GO:2000300	regulation of synaptic vesicle exocytosis (GO:2000300)	2443
HGNC:77	ABL2	ABL proto-oncogene 2, non-receptor tyrosine kinase	GO:0060074	synapse maturation (GO:0060074)	2674
HGNC:14290	NLGN2	neuroligin 2	GO:1905606	regulation of presynapse assembly (GO:1905606)	2745
HGNC:8008	NRXN1	neurexin 1	GO:0099560	synapse adhesion between pre- and post-synapse (GO:0099560)	2747
HGNC:8008	NRXN1	neurexin 1	GO:0099054	presynapse assembly (GO:0099054)	2748

HGNC:8008	NRXN1	neurexin 1	GO:1905606	regulation of presynapse assembly (GO:1905606)	2749
HGNC:14290	NLGN2	neuroligin 2	GO:0098698	postsynaptic specialization assembly (GO:0098698)	2754
HGNC:8008	NRXN1	neurexin 1	GO:0099150	regulation of postsynaptic specialization assembly (GO:0099150)	2757
HGNC:14290	NLGN2	neuroligin 2	GO:0098698	postsynaptic specialization assembly (GO:0098698)	2759
HGNC:12303	TRIO	trio Rho guanine nucleotide exchange factor	GO:0099170	postsynaptic modulation of chemical synaptic transmission (GO:0099170)	2844
HGNC:8148	OPHN1	oligophrenin 1	GO:0099149	regulation of postsynaptic neurotransmitter receptor endocytosis (GO:0099149)	2872
HGNC:8148	OPHN1	oligophrenin 1	GO:0098880	maintenance of postsynaptic specialization structure (GO:0098880)	2919
HGNC:7765	NF1	neurofibromin 1	GO:0098926	postsynaptic signaling pathway (GO:0098926)	3080
HGNC:7765	NF1	neurofibromin 1	GO:0099175	regulation of postsynapse organization (GO:0099175)	3081
HGNC:7765	NF1	neurofibromin 1	GO:0099159	regulation of modification of postsynaptic structure (GO:0099159)	3082
HGNC:1464	CAMK4	calcium/calmodulin dependent protein kinase IV	GO:0099170	postsynaptic modulation of chemical synaptic transmission (GO:0099170)	3669
HGNC:11411	CDKL5	cyclin dependent kinase like 5	GO:0050804	modulation of chemical synaptic transmission (GO:0050804)	3677

HGNC:11497	SYNGAP1	synaptic Ras GTPase activating protein 1	GO:0050804	modulation of chemical synaptic transmission (GO:0050804)	3681
HGNC:12496	UBE3A	ubiquitin protein ligase E3A	GO:0050804	modulation of chemical synaptic transmission (GO:0050804)	3729
HGNC:7648	NBEA	neurobeachin	GO:0098696	regulation of neurotransmitter receptor localization to postsynaptic specialization membrane (GO:0098696)	3780
HGNC:7648	NBEA	neurobeachin	GO:0050808	synapse organization (GO:0050808)	3781
HGNC:4845	HCN1	hyperpolarization activated cyclic nucleotide gated potassium channel 1	GO:0140232	intracellular cAMP-activated cation channel activity involved in regulation of presynaptic membrane potential (GO:0140232)	3800
HGNC:4845	HCN1	hyperpolarization activated cyclic nucleotide gated potassium channel 1	GO:0060078	regulation of postsynaptic membrane potential (GO:0060078)	3801
HGNC:8148	OPHN1	oligophrenin 1	GO:1900242	regulation of synaptic vesicle endocytosis (GO:1900242)	3804
HGNC:7648	NBEA	neurobeachin	GO:0050808	synapse organization (GO:0050808)	3833
HGNC:12363	TSC2	TSC complex subunit 2	GO:0099175	regulation of postsynapse organization (GO:0099175)	3928
HGNC:12303	TRIO	trio Rho guanine nucleotide exchange factor	GO:0050804	modulation of chemical synaptic transmission (GO:0050804)	3948

HGNC:10939	SLC1A1	solute carrier family 1 member 1	GO:0099170	postsynaptic modulation of chemical synaptic transmission (GO:0099170)	3959
HGNC:8008	NRXN1	neurexin 1	GO:0007416	synapse assembly (GO:0007416)	4049
HGNC:11444	STXBP1	syntaxin binding protein 1	GO:0099525	presynaptic dense core vesicle exocytosis (GO:0099525)	4072
HGNC:11079	SLC9A6	solute carrier family 9 member A6	GO:0099072	regulation of postsynaptic membrane neurotransmitter receptor levels (GO:0099072)	4101
HGNC:13830	CNTNAP2	contactin associated protein 2	GO:0099072	regulation of postsynaptic membrane neurotransmitter receptor levels (GO:0099072)	4191

Supplementary table 3: Table depicting the interactions between the molecules among different biological processes.

GeneMania analysis_interactions				
**Gene 1**	**Gene 2**	**Weight**	**Network group**	**Network**
CNTN5	SCN1A	0.000811414	Genetic Interactions	Lin-Smith-2010
CNTNAP2	CNTN5	0.000200683	Genetic Interactions	Lin-Smith-2010
CNTNAP2	CNR1	0.000258301	Genetic Interactions	Lin-Smith-2010
SYN1	CNTNAP2	0.001107931	Genetic Interactions	Lin-Smith-2010
SCN2A	CNTNAP2	0.000261679	Genetic Interactions	Lin-Smith-2010
SLC1A2	CNTN5	0.00048095	Genetic Interactions	Lin-Smith-2010
YWHAG	SCN1A	0.001266526	Genetic Interactions	Lin-Smith-2010
YWHAG	CNTN5	0.000551849	Genetic Interactions	Lin-Smith-2010
DPYSL2	SCN2A	0.001226949	Genetic Interactions	Lin-Smith-2010
STXBP1	YWHAG	0.001154238	Genetic Interactions	Lin-Smith-2010
STXBP1	DPYSL2	0.001968087	Genetic Interactions	Lin-Smith-2010
KCNMA1	CACNA1A	0.000787079	Genetic Interactions	Lin-Smith-2010
KCNMA1	SLC1A2	0.000409995	Genetic Interactions	Lin-Smith-2010
KCNMA1	STXBP1	0.000630381	Genetic Interactions	Lin-Smith-2010
SYN3	CNTNAP2	0.000188729	Genetic Interactions	Lin-Smith-2010

SYN3	SLC1A2	0.000452301	Genetic Interactions	Lin-Smith-2010
APBA1	CNR1	0.000716923	Genetic Interactions	Lin-Smith-2010
APBA1	SYN3	0.000523825	Genetic Interactions	Lin-Smith-2010
APBA2	CASK	0.001048023	Genetic Interactions	Lin-Smith-2010
NRXN2	CNTNAP2	0.000548867	Genetic Interactions	Lin-Smith-2010
SLC1A7	CNTN5	0.002963071	Genetic Interactions	Lin-Smith-2010
SLC1A3	OPHN1	0.000742923	Genetic Interactions	Lin-Smith-2010
SLC1A3	CNTNAP2	0.000241415	Genetic Interactions	Lin-Smith-2010
PACSIN1	SYN3	0.000758499	Genetic Interactions	Lin-Smith-2010
LAMB2	KCNMA1	0.00243188	Genetic Interactions	Lin-Smith-2010
SYTL3	CNTN5	0.001590342	Genetic Interactions	Lin-Smith-2010
SLC1A1	CACNA1A	0.000734548	Genetic Interactions	Lin-Smith-2010
SLC1A1	SYN3	0.00026452	Genetic Interactions	Lin-Smith-2010
SLC1A1	SLC1A3	0.000338363	Genetic Interactions	Lin-Smith-2010
SLC1A1	LAMB2	0.002269571	Genetic Interactions	Lin-Smith-2010
SLC1A1	SYTL3	0.001265236	Genetic Interactions	Lin-Smith-2010
DPYSL3	OPHN1	0.001500516	Genetic Interactions	Lin-Smith-2010
DPYSL3	CNTNAP2	0.000487596	Genetic Interactions	Lin-Smith-2010

DPYSL3	SLC1A3	0.001033361	Genetic Interactions	Lin-Smith-2010
DPYSL3	SLC1A1	0.000683407	Genetic Interactions	Lin-Smith-2010
SLC1A4	SCN2A	0.000798064	Genetic Interactions	Lin-Smith-2010
SCN3A	OPHN1	0.001058548	Genetic Interactions	Lin-Smith-2010
SCN3A	SLC1A3	0.000728991	Genetic Interactions	Lin-Smith-2010
SCN3A	PACSIN1	0.001382442	Genetic Interactions	Lin-Smith-2010
DYNLT1	CNTN5	0.002012648	Genetic Interactions	Lin-Smith-2010
DYNLT1	SCN2A	0.002624371	Genetic Interactions	Lin-Smith-2010
STX3	CNTNAP2	0.000431645	Genetic Interactions	Lin-Smith-2010
STX3	CASK	0.00142266	Genetic Interactions	Lin-Smith-2010
SCN5A	CNTN5	0.000733988	Genetic Interactions	Lin-Smith-2010
SCN5A	OPHN1	0.001282126	Genetic Interactions	Lin-Smith-2010
SCN5A	SYN3	0.000690267	Genetic Interactions	Lin-Smith-2010
SCN5A	SLC1A3	0.000882963	Genetic Interactions	Lin-Smith-2010
PLD1	SCN1A	0.001026327	Genetic Interactions	Lin-Smith-2010
PLD1	SCN2A	0.000583109	Genetic Interactions	Lin-Smith-2010
PLD1	SLC1A1	0.000355773	Genetic Interactions	Lin-Smith-2010
SYN1	SYN2	0.5	Shared protein domains	PFAM

CACNA1A	SCN1A	0.011216793	Shared protein domains	PFAM
SCN2A	SCN1A	0.06013327	Shared protein domains	PFAM
SCN2A	CACNA1A	0.011216793	Shared protein domains	PFAM
NRXN1	CNTNAP2	0.016034488	Shared protein domains	PFAM
KCNMA1	SCN1A	0.009878682	Shared protein domains	PFAM
KCNMA1	CACNA1A	0.01034484	Shared protein domains	PFAM
KCNMA1	SCN2A	0.009878682	Shared protein domains	PFAM
SYN3	SYN2	0.5	Shared protein domains	PFAM
SYN3	SYN1	0.5	Shared protein domains	PFAM
APBA2	APBA1	0.026574994	Shared protein domains	PFAM
NRXN2	CNTNAP2	0.023281574	Shared protein domains	PFAM
NRXN2	NRXN1	0.040059816	Shared protein domains	PFAM
SLC1A7	SLC1A2	0.16666667	Shared protein domains	PFAM
SLC1A3	SLC1A2	0.16666667	Shared protein domains	PFAM
SLC1A3	SLC1A7	0.16666667	Shared protein domains	PFAM
SLC1A6	SLC1A2	0.16666667	Shared protein domains	PFAM
SLC1A6	SLC1A7	0.16666667	Shared protein domains	PFAM
SLC1A6	SLC1A3	0.16666667	Shared protein domains	PFAM

SLC1A1	SLC1A2	0.16666667	Shared protein domains	PFAM
SLC1A1	SLC1A7	0.16666667	Shared protein domains	PFAM
SLC1A1	SLC1A3	0.16666667	Shared protein domains	PFAM
SLC1A1	SLC1A6	0.16666667	Shared protein domains	PFAM
DPYSL3	DPYSL2	0.1044308	Shared protein domains	PFAM
SLC1A4	SLC1A2	0.16666667	Shared protein domains	PFAM
SLC1A4	SLC1A7	0.16666667	Shared protein domains	PFAM
SLC1A4	SLC1A3	0.16666667	Shared protein domains	PFAM
SLC1A4	SLC1A6	0.16666667	Shared protein domains	PFAM
SLC1A4	SLC1A1	0.16666667	Shared protein domains	PFAM
SCN3A	SCN1A	0.060133267	Shared protein domains	PFAM
SCN3A	CACNA1A	0.011216793	Shared protein domains	PFAM
SCN3A	SCN2A	0.060133267	Shared protein domains	PFAM
SCN3A	KCNMA1	0.009878681	Shared protein domains	PFAM
SLC1A5	SLC1A2	0.16666667	Shared protein domains	PFAM
SLC1A5	SLC1A7	0.16666667	Shared protein domains	PFAM
SLC1A5	SLC1A3	0.16666667	Shared protein domains	PFAM
SLC1A5	SLC1A6	0.16666667	Shared protein domains	PFAM

SLC1A5	SLC1A1	0.16666667	Shared protein domains	PFAM
SLC1A5	SLC1A4	0.16666667	Shared protein domains	PFAM
DPYSL4	DPYSL2	0.1044308	Shared protein domains	PFAM
DPYSL4	DPYSL3	0.1044308	Shared protein domains	PFAM
SCN5A	SCN1A	0.060133267	Shared protein domains	PFAM
SCN5A	CACNA1A	0.011216793	Shared protein domains	PFAM
SCN5A	SCN2A	0.060133267	Shared protein domains	PFAM
SCN5A	KCNMA1	0.009878681	Shared protein domains	PFAM
SCN5A	SCN3A	0.060133263	Shared protein domains	PFAM
SYN1	SYN2	0.36995652	Shared protein domains	INTERPRO
CACNA1A	SCN1A	0.009905666	Shared protein domains	INTERPRO
SCN2A	SCN1A	0.05906199	Shared protein domains	INTERPRO
SCN2A	CACNA1A	0.007851321	Shared protein domains	INTERPRO
NRXN1	CNTNAP2	0.021636711	Shared protein domains	INTERPRO
KCNMA1	SCN1A	0.010794256	Shared protein domains	INTERPRO
KCNMA1	CACNA1A	0.011104608	Shared protein domains	INTERPRO
SYN3	SYN2	0.36995652	Shared protein domains	INTERPRO
SYN3	SYN1	0.36995652	Shared protein domains	INTERPRO

APBA2	APBA1	0.029106779	Shared protein domains	INTERPRO
NRXN2	CNTNAP2	0.023267513	Shared protein domains	INTERPRO
NRXN2	NRXN1	0.041728187	Shared protein domains	INTERPRO
SLC1A7	SLC1A2	0.16666667	Shared protein domains	INTERPRO
SLC1A3	SLC1A2	0.16666667	Shared protein domains	INTERPRO
SLC1A3	SLC1A7	0.16666667	Shared protein domains	INTERPRO
SLC1A6	SLC1A2	0.16666667	Shared protein domains	INTERPRO
SLC1A6	SLC1A7	0.16666667	Shared protein domains	INTERPRO
SLC1A6	SLC1A3	0.16666667	Shared protein domains	INTERPRO
SLC1A1	SLC1A2	0.16666667	Shared protein domains	INTERPRO
SLC1A1	SLC1A7	0.16666667	Shared protein domains	INTERPRO
SLC1A1	SLC1A3	0.16666667	Shared protein domains	INTERPRO
SLC1A1	SLC1A6	0.16666667	Shared protein domains	INTERPRO
DPYSL3	DPYSL2	0.083236896	Shared protein domains	INTERPRO
SLC1A4	SLC1A2	0.16666667	Shared protein domains	INTERPRO
SLC1A4	SLC1A7	0.16666667	Shared protein domains	INTERPRO
SLC1A4	SLC1A3	0.16666667	Shared protein domains	INTERPRO
SLC1A4	SLC1A6	0.16666667	Shared protein domains	INTERPRO

SLC1A4	SLC1A1	0.16666667	Shared protein domains	INTERPRO
SCN3A	SCN1A	0.05475821	Shared protein domains	INTERPRO
SCN3A	CACNA1A	0.007279205	Shared protein domains	INTERPRO
SCN3A	SCN2A	0.04957733	Shared protein domains	INTERPRO
SLC1A5	SLC1A2	0.16666667	Shared protein domains	INTERPRO
SLC1A5	SLC1A7	0.16666667	Shared protein domains	INTERPRO
SLC1A5	SLC1A3	0.16666667	Shared protein domains	INTERPRO
SLC1A5	SLC1A6	0.16666667	Shared protein domains	INTERPRO
SLC1A5	SLC1A1	0.16666667	Shared protein domains	INTERPRO
SLC1A5	SLC1A4	0.16666667	Shared protein domains	INTERPRO
DPYSL4	DPYSL2	0.083236896	Shared protein domains	INTERPRO
DPYSL4	DPYSL3	0.083236896	Shared protein domains	INTERPRO
SCN5A	SCN1A	0.07446225	Shared protein domains	INTERPRO
SCN5A	CACNA1A	0.009905666	Shared protein domains	INTERPRO
SCN5A	SCN2A	0.05906199	Shared protein domains	INTERPRO
SCN5A	KCNMA1	0.010794256	Shared protein domains	INTERPRO
SCN5A	SCN3A	0.05475821	Shared protein domains	INTERPRO

2 Supplementary Figures

Supplementary figure 1:

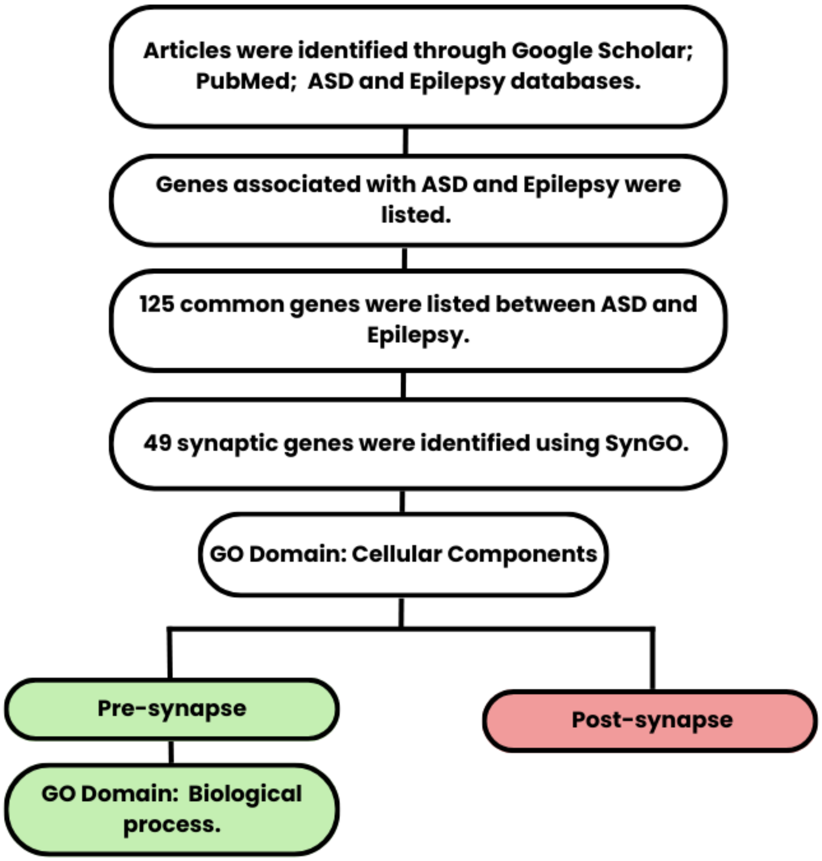

Supplementary Figure 1. Schematic representation of methodology followed in this study.

Supplementary figure 2:

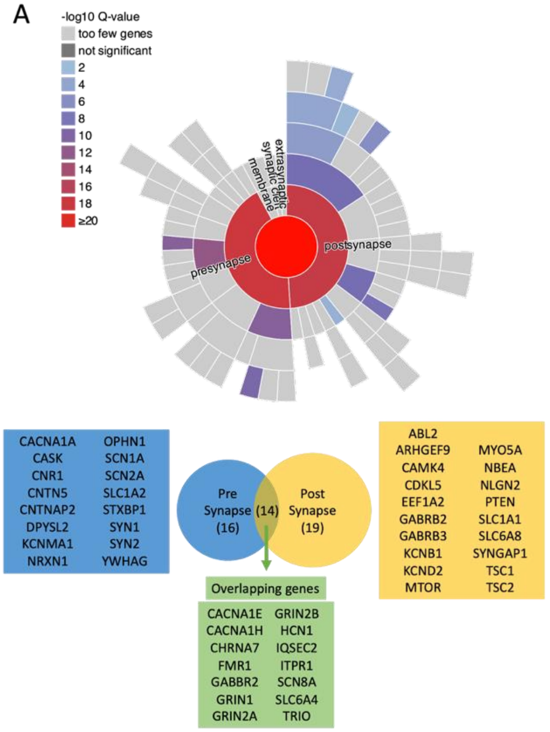

B

Ontology term	Enrichment domain	Gene count	p-value	List of genes
Presynapse	Cellular Component	16	2.66e-23	*CASK; DPYSL2; YWHAG; OPHN1; STXBP1; CACNA1A; KCNMA1; NRXN1; SYN1; SYN2; CNTN5; SCN2A; CNR1; SCN1A; SLC1A2; CNTNAP2*
Presynaptic membrane	Cellular Component	8	6.03e-13	*CNTN5; STXBP1; SCN2A; CNR1; NRXN1; SCN1A; SLC1A2; CNTNAP2*
Integral component of presynaptic membrane	Cellular Component	6	1.23e-9	*SCN2A; CNR1; NRXN1; SCN1A; SLC1A2; CNTNAP2*
Presynaptic active zone	Cellular Component	4	2.50e-6	*STXBP1; CACNA1A; KCNMA1; NRXN1*
Integral component of presynaptic active zone membrane	Cellular Component	3	1.46e-5	*CACNA1A; KCNMA1; NRXN1*

Supplementary Figure 2. (A) Sunburst image depicts gene enrichment analyses for common synaptic genes in ASD and epilepsy. Enriched gene ontology (GO) terms related to cellular components, depicting the synaptic localization of the genes. Venn diagram illustrating overlapping genes and specific pre- and post-synaptic genes between both disorders, categorized by cellular component GO terms. (B) Summary of the SynGO gene ontology database, categorizing gene products based on their cellular components. Genes or proteins associated with cellular structures like transmembrane or extracellular membranes are classified under "cellular components".
